# Phytoplankton diversity and size structure in the Central-Southern Tyrrhenian Sea: implications for microbial functioning

**DOI:** 10.1007/s00248-025-02650-w

**Published:** 2025-11-20

**Authors:** Carmela Caroppo, Gabriella Caruso, Alessandro Bergamasco, Franco Decembrini

**Affiliations:** 1 National Research Council, Water Research Institute, (CNR-IRSA), Section of Taranto, Taranto, Italy; 2 NBFC—National Biodiversity Future Center, Palermo, Italy; 3 National Research Council, Institute of Polar Sciences, (CNR-ISP), Section of Messina, Messina, Italy; 4 National Research Council, Institute of Marine Sciences (CNR-ISMAR), Section of Venice, Castello 2737/F, 30122 Venice, Italy

**Keywords:** Phytoplankton, Diversity, Primary production, Enzymes, Trophic size-structure, Tyrrhenian Sea

## Abstract

Microbial community dynamics in relation to mesoscale hydrographical features are almost unknown particularly in the pelagic Central-Southern Tyrrhenian Sea. To get a more comprehensive view of phytoplankton community structure and microbial community functioning, datasets of phytoplankton abundance, composition and some microbial enzyme activities (leucine aminopeptidase, LAP, beta-glucosidase, GLU and alkaline phosphatase, AP) from six cruises carried out twenty years ago were analyzed. Hydrographic characteristics identified the presence of both Atlantic Waters (AW) and Tyrrhenian Intermediate Waters (TIW). Size structure of phytoplankton biomass showed an unexpected high contribution of the pico-phytoplankton to the total primary production (> 60%) determining a predominant microbial food web. Phytoplankton distribution patterns varied more significantly on a seasonal rather than spatial scale. Autumn assemblages were characterized by the highest abundance and carbon content, with species mainly belonging to dinoflagellates whose growth was supported by intense microbial activities. In contrast, in the summer diatoms developed in unstable TIW where microbial activity was declining. Enzymatic activities varied in the different water masses and seasons, with high LAP activity in summer AW (s-AW) as well as in deep TIW (d-TIW), while AP and GLU reached their maximum in autumn AW (a-AW), suggesting quick organic matter recycling. Coupled primary production and hydrolysis in mixed AW (m-AW) and in a-AW indicated synchronized autotrophic and heterotrophic processes, while in TIW organic matter was only partially recycled. Overall, microbial metabolism was closely shaped by hydrographic and seasonal dynamics, confirming its key role in biogeochemical cycles. Our data could provide a baseline study for future research dealing with the microbial functioning in this Mediterranean region.

**Supplementary Information:**

The online version contains supplementary material available at 10.1007/s00248-025-02650-w.

## Introduction

Microbial plankton is crucial in marine ecosystems, driving biogeochemical cycles, which influence all the trophic levels [1]. Phytoplankton community contributes to about half of global net primary production, playing a key role in ocean–atmosphere interactions [2, 3]. Its diversity and spatial–temporal variation affect marine ecosystem dynamics [4]. Climate change is altering phytoplankton communities reducing evenness, reshaping assemblages, and impacting productivity and food webs [5]. Phytoplankton size structure shapes marine food webs, with two main pathways: the herbivorous food web (dominated by large phytoplankton and zooplankton) and the microbial loop (involving microbes and protozoa). Transitions between these pathways influence whether carbon is exported in deeper layers or recycled within the upper waters. Continuous trophic pathway includes multivorous and microbial webs that blend both systems, each playing distinct roles in carbon cycling [6]. The balance between small and large phytoplankton is regulated by environmental factors like water column stability, nutrient availability, and zooplankton grazing [7–10].

Heterotrophic microbes play key roles by breaking down organic polymers via enzymatic hydrolysis, that allows organic matter turnover [4]. Only small molecules can be directly uptaken [11]; enzymes like leucine aminopeptidase (LAP), beta-glucosidase (GLU) and alkaline phosphatase (AP) help degrading proteins, polysaccharides and organic phosphates, respectively, supporting carbon, nitrogen and phosphorus cycles [12].

Hydrographic features strongly influence microbial communities, but few *in-situ* studies address their diversity and function in the Mediterranean Sea [13, 14]. Due to its semi-enclosed nature, low water residence time, and sensitivity to climate change, this basin is suitable for such studies [14, 15,16 and references therein]. Increased stratification, driven by rising sea surface temperatures, affects phytoplankton biomass, bloom timing, cell size, and taxonomic composition [17–19]. In the oligotrophic Central-Southern Tyrrhenian Sea, stronger bottom-up control could further limit photosynthetic activity [20]. While surface chlorophyll-*a* data show no clear long-term trends [21], deeper water column processes remain understudied.

Mesoscale features can influence microbial and phytoplankton communities, with significant implications for the synchronization of autotrophic and heterotrophic processes. These features are also expected to modulate microbial contributions to biogeochemical cycling in temperate aquatic systems. Using an extended dataset collected two decades ago, this study investigates the relationships between hydrographic processes and the functioning of phytoplankton in terms of abundance, diversity, size-structure, activity, and microbial metabolism across seasons and depths. Our focus is to investigate which phytoplankton assemblages are hosted and which trophic pathways are active in the different layers of the euphotic zone. The final goal is to build a baseline knowledge on the role of microbial compartment within the Central-Southern Tyrrhenian ecosystem functioning to support studies on future environmental scenarios in the Mediterranean Sea.

## Materials and Methods

### Study area

Inputs of Atlantic origin from the western Mediterranean enter the surface layer of the Tyrrhenian Sea through the Sicily-Sardinia channel, mainly at its eastern side [22]. The Atlantic Waters (AW) move along the northern coast of Sicily and form a large cyclonic flow that laps all the western Italian coast and exits through the Strait of Corsica where it feeds the Ligurian-Provençal current along the northern border of the western Mediterranean Sea [23]. During its transit within the Tyrrhenian basin, the branch of Atlantic origin is strongly modified (Modified Atlantic Waters, MAW) by mixing with the underlying waters of Levantine origin that enter the basin from the Sicilian Channel through a shallow stretch off the coast of the Egadi Islands. Surface waters of the Tyrrhenian Sea, formerly called Tyrrhenian Surface Waters (TSW), are now renamed AW as well as MAW; while the mixed waters of the Tyrrhenian Sea, above the Levantine Intermediate Waters (LIW), are called Tyrrhenian Intermediate Waters (TIW) [23].

### Experimental Dataset and Hydrographical Parameters

**Fig. 1:**
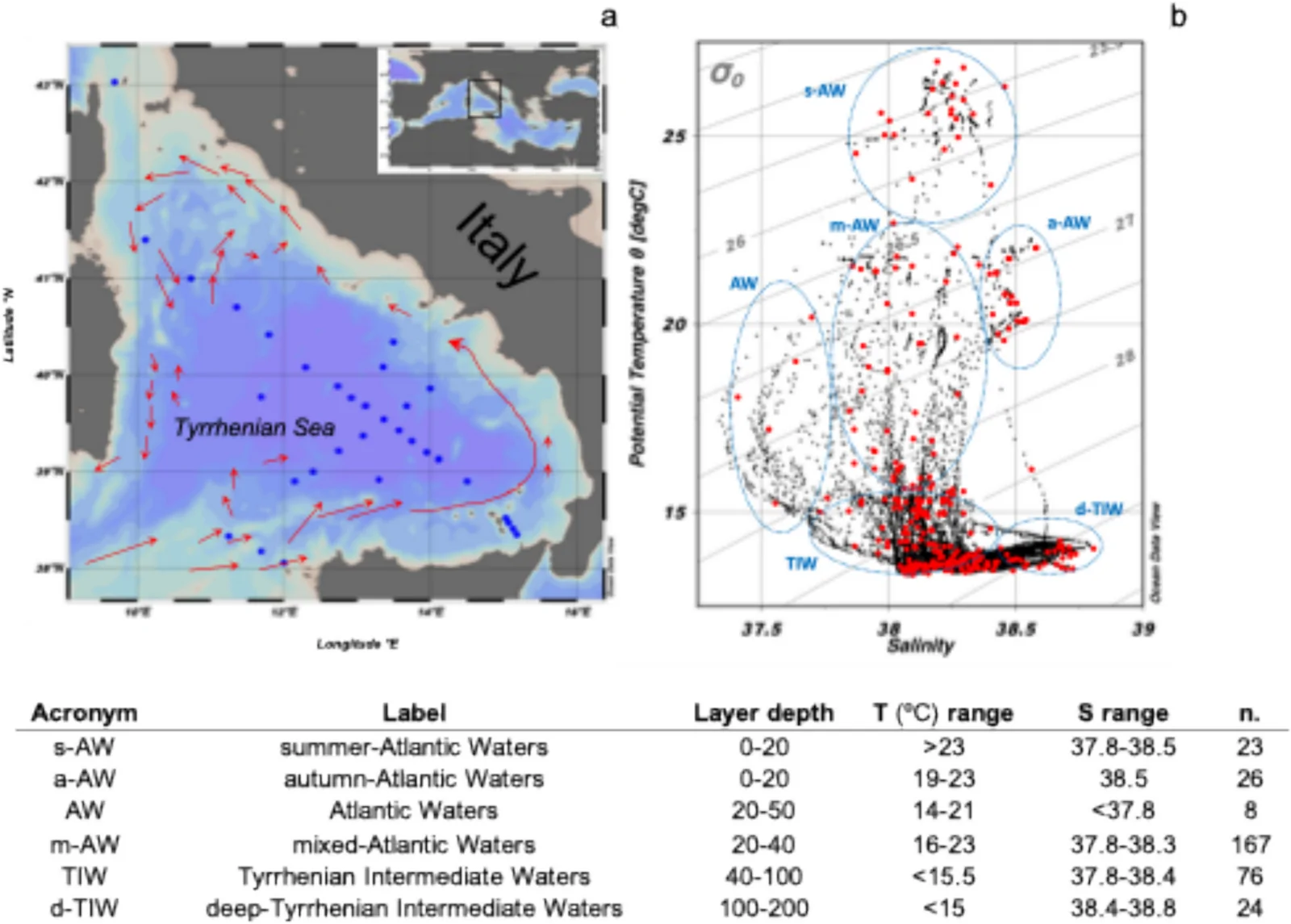
**a**) Sampling stations in the Central-Southern Tyrrhenian Sea during the six seasonal cruises and surface currents (red arrows); **b**) θ-S diagram of the CTD casts (0–200 m) in the study area with indication of the identified water masses. Red dots are the positions of the samples (324 in total). The labels and acronyms of the water masses identified in the study area, along with their respective layer depths, temperature and salinity ranges, and the number of samples collected for each water mass, are reported below

This study gathers physical–chemical and biological datasets collected during six oceanographic cruises covering different seasons between spring 2005 and autumn 2006 and were partially already published [24, 25]. To make results comparable, sampling strategies and analytical methods were consistent during all the cruises. Samplings were carried out in the Central-Southern Tyrrhenian Sea (43° N – 9.0° E and 38° N–15.5° E) at sixty-five stations mainly located on two NW-to-SE and SW-to-NE transects (Fig. 1 a). Temperature (T) and salinity (S) vertical profiles were recorded by using a SeaBird 911plus CTD-O-Fl profiler up to the maximum depth of each station. For this study only CTD data in the layer 0–200 m were considered. For the collection of samples, a Rosette sampler with 10 L Niskin bottles was used. The water sampling was carried out at 4–5 depths in the photic layer (0–120 m) of the water column.

### Size-fractionated Chlorophyll-*a* and Primary Production Rates

Chlorophyll-*a* (chl-*a*) and degraded pigments (phaeopigments, phaeo) were measured fluorimetrically (n = 307 samples). Different phytoplankton size-fractions (micro- > 10.0 μm; nano- 2.0–10.0 μm; and pico-phytoplankton, 0.2–2.0 μm) were sequentially filtered. The chl-*a* and phaeo were extracted, for 24 h at 4 °C, with 90% acetone from the homogenized filter and determined with a Varian (mod. Cary Eclipse) spectrofluorometer [26]. Total and size-fractionated primary production (PP) were measured with the standard ^14^C labelling technique (n = 161 samples). Details of the procedures can be found in Decembrini et al. [26].

Trophic pathways were assessed according to Legendre and Rassoulzadegan [6].

### Phytoplankton Abundance and Species Composition

Water samples (500 mL) collected for the phytoplankton analysis were preserved in dark glass bottles with an acid Lugol’s iodine solution (final dilution 1.0%) and maintained at 4 °C until laboratory analysis. A total of 129 samples was analysed. An inverted microscope (Labovert FS Leitz) equipped with phase contrast was used for identification and counting which were performed according to the Utermöhl method [27]. Following a preliminary assessment of phytoplankton abundance, subsamples ranging from 50 to 100 mL were settled in Utermöhl chambers and analyzed under magnifications of 400 × and 630 ×. Phytoplankton counts were conducted along 1–4 transects or within 30–60 randomly selected fields. Additionally, half of the chamber was examined at 200 × magnification to include less abundant taxa. A minimum of 200 cells was counted per sample to ensure a 14% confidence limit [28], which is generally considered acceptable. Phytoplankton cell sizes were assessed using the digital camera AXIOCAM ICc 5 (Carl Zeiss, Oberkochen, Germany). Biovolume was determined by assigning to each cell one geometrical body or, in some cases, to a combination of more geometrical bodies, and applying standard formulae [29]. Carbon content was calculated using the conversion factors suggested by Menden-Deuer and Lessard [30].

### Microbial Activities in Organic Matter Decomposition

Microbial decomposition of organic matter was assessed through enzyme activity using fluorogenic substrates. L-leucine-MCA, MUF-β-D-glucopyranoside, and MUF-phosphate (Sigma-Aldrich) were used to measure LAP, B-GLU, and AP activities. Substrates (20–200 nmol L⁻^1^ final concentration) were added to 10 mL seawater samples. Fluorescence from substrate hydrolysis was measured at time zero and after 3 h of incubation at *in situ* temperature using a Turner TD-700 fluorimeter. Maximum hydrolysis rates (Vmax) were expressed as nmol of leucine, glucoside, or phosphate released per liter per hour [31].

### Statistical Analysis

All the available CTD raw data (65 profiles, in the 0–120 m layer) were quality checked and processed with Ocean Data View (ODV v. 5.5.2.) software [32] to calculate derived variables and obtain T/S diagram. A preliminary automatic clusterization guided the assignment of the discrete water samples to six clusters.

Spatial distribution of the phytoplankton community was analysed using the PRIMER v.7 software (Primer-E) [33]. Statistical comparisons among water masses and seasons were obtained by non-parametric multidimensional scaling (nMDS) performed on Bray–Curtis similarity matrices (four-root transformed data) [33]. Differences in the phytoplankton community assemblages between different water masses and seasons were evaluated by one-way Analysis of Similarities (ANOSIM). Similarity percentage (SIMPER) measured the contribution of each species/morpho-species to the Bray–Curtis similarity between the groups of samples.

The co-occurrence matrix of species/taxa was used to build the established network. Only taxa appearing in at least 15 samples were considered, as a good compromise between the network detail, computational effort and possible noise. To calculate Spearman’s rank coefficient the R package “Hmisc” was used [34]. To detect the sub-community structure of the network and assign the membership to each taxon, the Fast-Greedy algorithm (Louvain method) was applied [35]. Local centrality metrics [36] (i.e., degree, clustering, closeness and betweenness) allowed the comparison of node topology across relevant groups of taxa (ANOVA, K_W test) and gave insight on the importance of species/taxa within the phytoplankton community. R “igraph” and Gephi software packages [37] were used to calculate the metrics and visualize the networks, respectively.

## Results

### Hydrographical Parameters

The water column features of the 0–200 m layer are represented in the *θ* θ-S diagram (Fig. 1 b). The surface layer is characterized by the presence of Atlantic Waters (AW) subject to different mixing processes throughout the seasons. The surface summer waters (s-AW) exhibit a temperature > 23 °C; in autumn and mainly in the Eastern sub-region the surface layer hosts slightly saltier waters (a-AW) that are cooling down while the summer inflow of AW is in the subsurface layer of Western sub-region. Surface mixed waters (m-AW) have intermediate temperature and salinity signature. Just below (40–200 m), the Tyrrhenian Intermediate Water (TIW) is formed by mixing between AW and the underlying Levantine Intermediate Waters (LIW); it can be divided into an upper part which is still affected by seasonal surface warming processes where the Deep Chlorophyll Maximum (DCM) is located and the deep TIW (d-TIW) saltier and less-thermally-variable lower part. The averaged values of the main environmental parameters are reported in Table S1.

### Distribution of Fractionated Chlorophyll-*a*, Primary Production and Assimilation Number

The integrated chl-*a* concentration in the euphotic layer (0–120 m) averaged 8.5 ± 3.2 mg m⁻^2^, with values ranging from 2.3 to 16.8 mg m⁻^2^. Along the North–South transect (average 8.2 ± 4.1 mg m⁻^2^), local peaks were associated with known mesoscale features. The phytoplankton community was dominated by pico-phytoplankton (66%), followed by nano- (21%) and micro-phytoplankton (13%). The integrated phaeopigments were approximately one-third of active chl-*a*, and their size distribution mirrored that of chl-*a*.

A strong linear correlation (R^2^ = 0.93) was observed between pico-phytoplankton and total chl-*a*. At low chl-*a* concentrations (< 0.2 mg m⁻^3^), nano-phytoplankton showed weaker correlations, while micro-phytoplankton contributed significantly to chl-*a* when concentrations exceeded 0.2 mg m⁻^3^, particularly in d-TIW and m-AW during autumn.

**Fig. 2:**
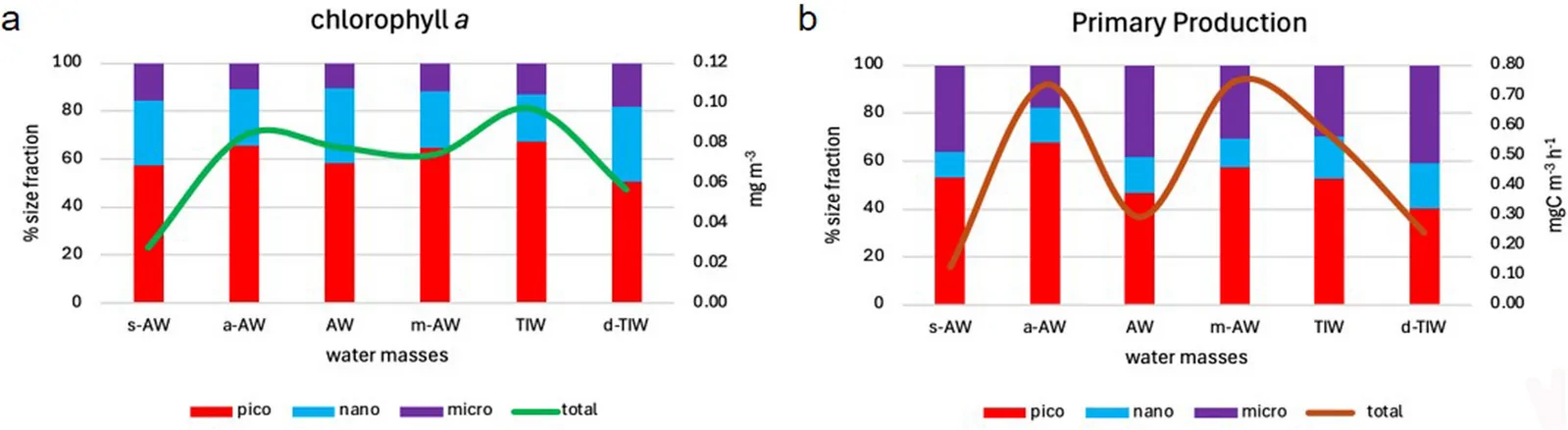
Average integrated chlorophyll-*a* (**a**) and primary production (**b**) distribution (line) along the euphotic layer and percentage of size-fractionated phytoplankton community (histogram) in the six water masses

Vertical chl-*a* distribution (Fig. 2 a) revealed a DCM between 50–75 m in TIW, absent in winter when chl-*a* was confined to the surface (0–50 m). At the DCM, chl-*a* averaged 0.125 ± 0.069 mg m⁻^3^, with lower concentrations above (0.053 mg m⁻^3^) and below (0.047 mg m⁻^3^). The DCM size community was mainly composed of pico-phytoplankton (69%), with nano- and micro- fractions contributing 18% and 13%, respectively. Above the DCM, nano-phytoplankton increased to 23%, while pico-phytoplankton decreased to 59%.

Primary production (PP in 28 stations) in the photic layer (up to 0.1% E_0_^+^) averaged 52.2 ± 35.4 mgC m⁻^2^ h⁻^1^, ranging from 12.5 to 136.1 mgC m⁻^2^ h⁻^1^. PP was primarily driven by pico-phytoplankton (60%), followed by micro- (30%) and nano- (10%) fractions (Fig. 2 b). Total PP correlated strongly with pico-phytoplankton (R^2^ = 0.91), while nano-phytoplankton maintained a constant rate (0.2 mgC m⁻^3^ h⁻^1^) regardless of total PP. Micro-phytoplankton became more active when PP exceeded 0.2 mgC m⁻^3^ h⁻^1^, especially in TIW, replacing the nano-fraction.

Vertical PP distribution showed different average rates in the upper DCM (m-AW) and DCM (TIW) layers: 0.97 ± 0.74 and 0.57 ± 0.70 mgC m⁻^3^ h⁻^1^, respectively. Below the DCM (d-TIW), PP dropped to 0.30 ± 0.40 mgC m⁻^3^ h⁻^1^. At the a-AW, pico-phytoplankton contributed 65% to total PP, consistent with chl-*a* patterns. In upper (AW) and lower (d-TIW) layers, pico-phytoplankton decreased, with micro-phytoplankton rising to 40%.

**Fig. 3:**
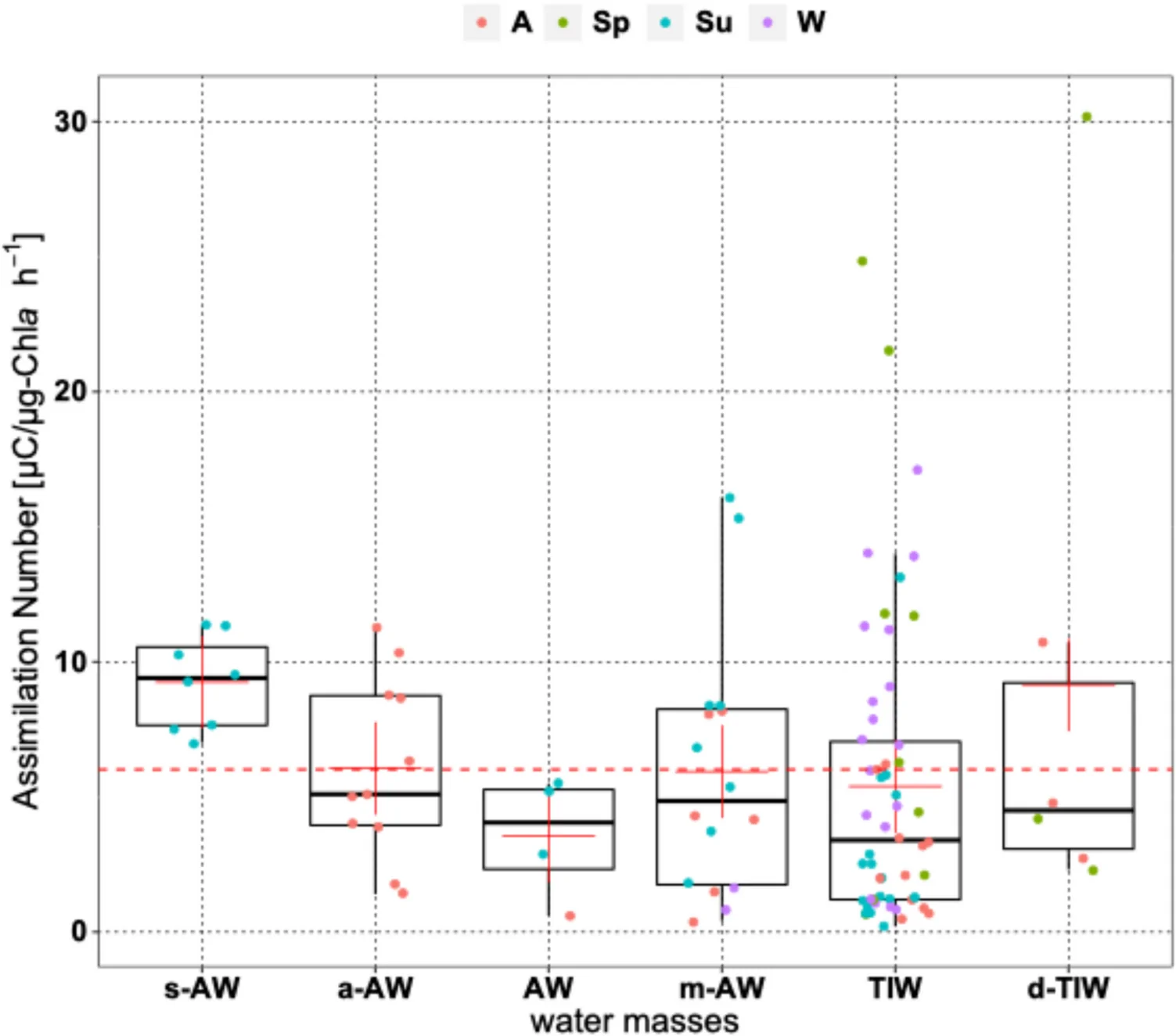
Box plots of the Assimilation Number (AN) across different water masses. Red crosses represent the mean values for each group. The horizontal dotted line indicates the overall mean. Seasons are abbreviated as follows: A = Autumn; Sp = Spring; Su = Summer; W = Winter

The Assimilation Number (AN), representing PP per unit chl-*a*, averaged 6.5 ± 11.1 mgC (mg chl-*a*)⁻^1^ h⁻^1^. AN peaked in spring, declined in autumn, and was lowest in summer. Size fractions followed similar trends, with nano-phytoplankton showing the lowest AN. In s-AW, AN differed notably from the other water masses (Fig. 3).

**Fig. 4:**
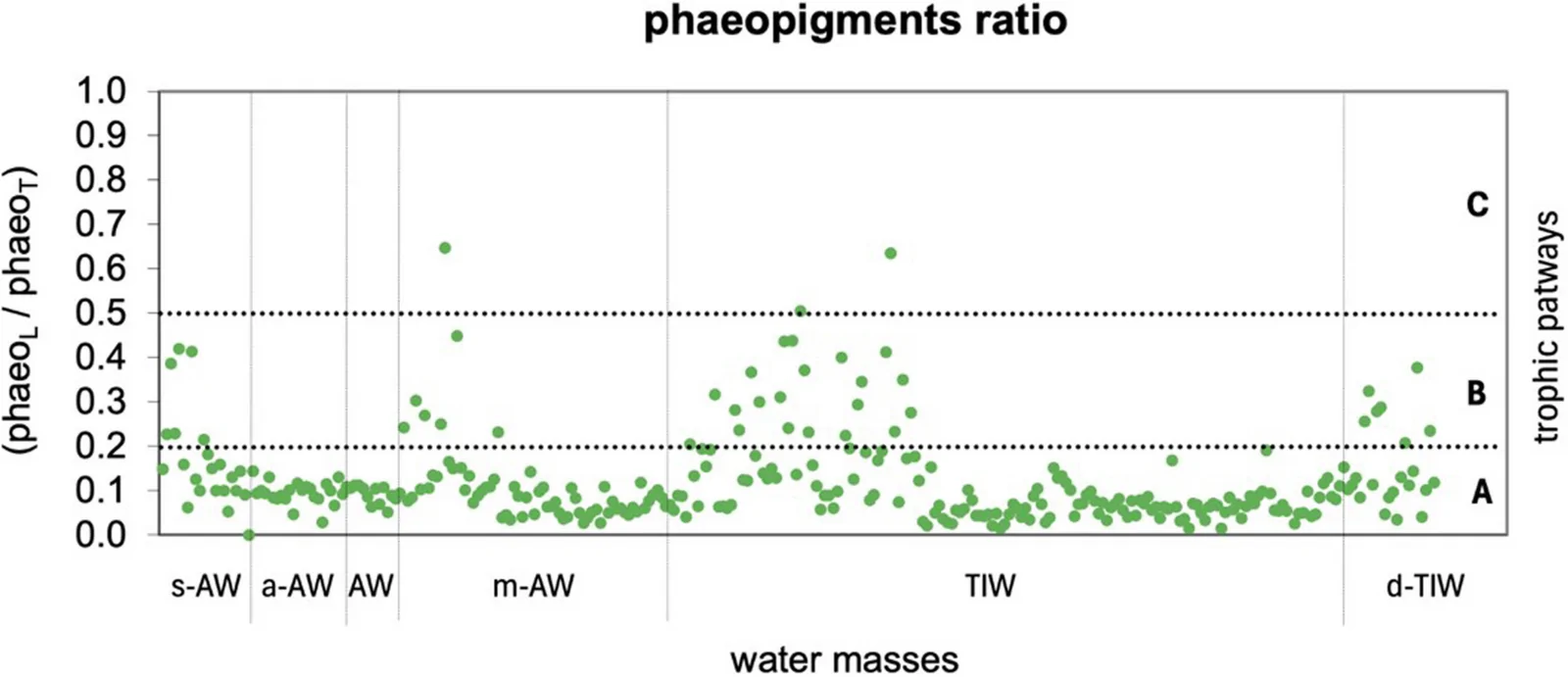
Trophic pathways in the six water masses, assessed using the ratio of micro-phytoplankton to total phaeopigments (phaeo_L_ = Large > 10 μm; phaeo_T_ = Total > 0.2 μm). According to the *continuum* trophic pathway concept (*sensu *Legendre & Rassoulzadegan [6]), the phaeo_L_/phaeo_T_ ratio defines the trophic mode: < 0.2 indicates a microbial pathway (**A**); 0.2–0.5 indicates a multivorous pathway (**B**); > 0.5 indicates an herbivorous pathway (**C**)

Microbial trophic pathways were assessed using phaeopigment ratios between Large-sized (L) and Total (T) phytoplankton; microbial pathways dominated across water masses, particularly in a-AW and AW (Fig. 4). Multivorous conditions (0.2 < phaeo_L_/phaeo_T_ < 0.5) were identified in TIW, d-TIW, and s-AW. In m-AW, microbial dominance persisted, although some summer-autumn ratios indicated multivorous or even herbivorous activity (> 0.5).

## Phytoplankton Abundance and Composition

Phytoplankton abundance and biomass varied widely across the study area, ranging from 2.01 to 90.40 × 10^3^ cells L⁻^1^ and from 0.11 to 84.39 µg C L⁻^1^, respectively. As shown in Fig. S1a & b, slightly elevated mean values were observed in the s-AW, m-AW, and TIW. In terms of abundance, the community was dominated by “other phytoflagellates” (Fig. S1c) which accounted for 62.3 ± 9.5% of total cells. Dinoflagellates and diatoms followed with 19.5 ± 6.2% and 10.9 ± 8.4%, respectively, while coccolithophorids were the least abundant (7.2 ± 2.6%). Despite their lower abundance, dinoflagellates were the primary contributors to the phytoplankton carbon pool (Fig. S1d), representing 71.0 ± 14.5% of the total amount. Diatoms contributed 22.5 ± 11.4%, while “other phytoflagellates” and coccolithophorids accounted for 5.4 ± 4.2% and 1.1 ± 0.7%, respectively.

Seasonal patterns revealed that phytoplankton abundance and carbon content peaked during the summer months (Fig. S2). Diatom abundance percentages (Fig. S2a & b) reached their highest values in summer, while those of dinoflagellates peaked in spring and summer, coccolithophorids in spring, and nano-sized phytoflagellates during the autumn–winter period. Regarding the percentage contribution of the different groups to the total phytoplankton biomass (Fig. S2c & d), dinoflagellates were consistently dominant, especially in spring and summer, while diatoms reached their peak in summer.

A total of 183 taxa were identified, including 70 diatoms, 103 dinoflagellates, 5 coccolithophores, and 5 species classified as “other phytoflagellates” (Table S2).

Species composition showed limited spatial variability across water masses (ANOSIM, R = 0.136, *p* = 0.5%), while seasonality significantly influenced phytoplankton assemblages (ANOSIM, R = 0.313, *p* = 0.1%). TIW exhibited distinct species composition compared to s-AW and m-AW, with higher diatom abundance (Fig. S1c) and diversity (SIMPER, Average Dissimilarity: 66.34% *vs*. s-AW and 64.18% *vs*. m-AW). In comparison with the other water masses, the specific composition of the communities did not differ significantly (SIMPER analysis; average dissimilarity < 50%). Assemblages in s-AW and m-AW shared common features (AD < 60%), with dinoflagellates dominating and diatoms forming the secondary group, slightly more prevalent in m-AW (Fig. S1c). Undetermined nano-sized phytoflagellates represented the common trait across all water masses, contributing to the lack of significant compositional differences.

**Fig. 5:**
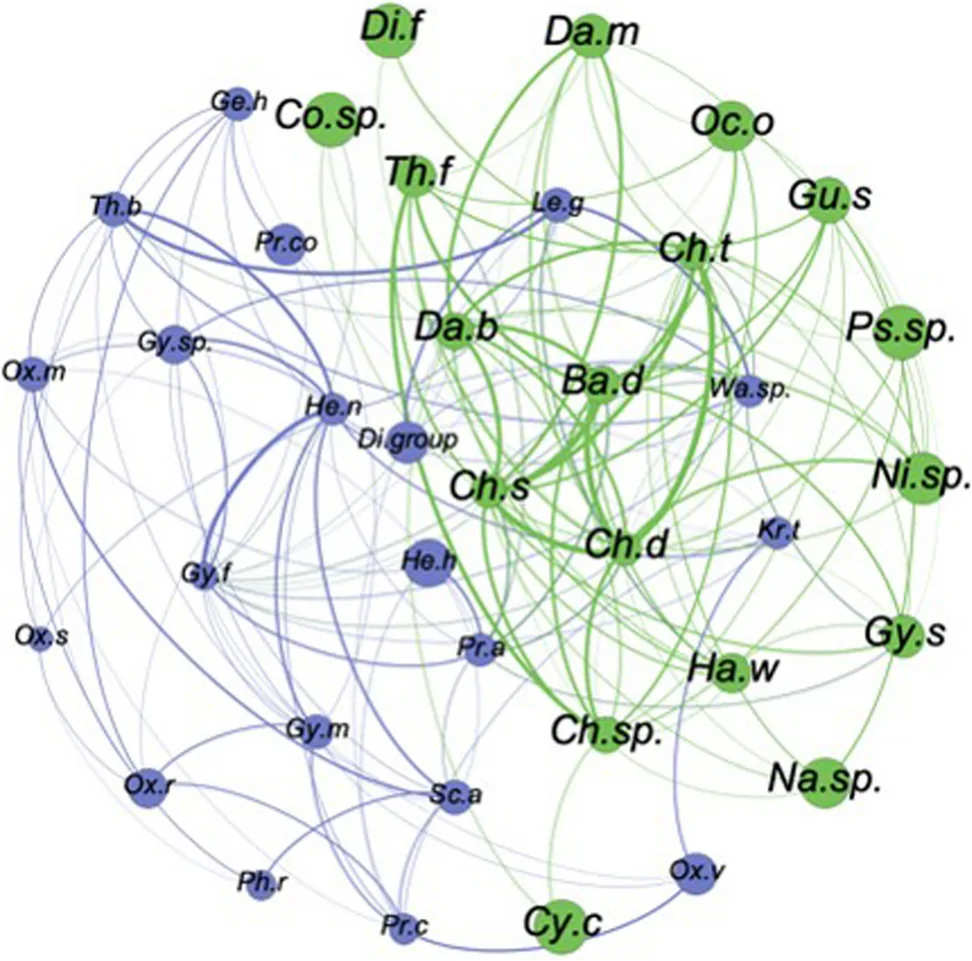
Phytoplankton community network. Significant co-occurrence of taxa. A link (edge) is established between two taxa (nodes) when their Spearman’s rank coefficient is positive (r > 0.1) and significant (*p *< 0.05). Colors of nodes encode the membership to a sub-community (green: mainly micro-sized diatoms; blue: mixotrophic dinoflagellates). Thickness of edges is proportional to the strength of correlation

SIMPER results were supported by dispersion index analysis [34], which failed to clearly distinguish “core” and “occasional” taxa across samples and sub-groups (water masses, seasons). A set of 38 significantly co-occurring taxa was identified, comprising 18 diatoms, 17 dinoflagellates, 2 dictyophyceans, and 1 coccolithophorid. Modularity analysis identified two sub-communities that included taxa sharing functional affinity and convergence of traits (Fig. 5, Table 1).

**Table 1:** Species list presented in the network of co-occurrences. Size class, trophy and phylum are indicated. Topological features and sub-community membership of each taxon/node are shown

Label	Species name	Size Class	Trophy	Taxonomy	degree	Memb Fast-Greedy	part_coeff	z_score
Co.sp.	*Coscinodiscus* sp.	M	A	DIAT	3	1	0.44	−1.47
Di.f	*Dictyocha fibula* Ehrenb.	M	A	DICTYO	2	1	0	−1.47
Th.f	*Thalassionema frauenfeldii *(Grunow) Temp.& Perag.	M	A	DIAT	9	1	0.2	0.03
Ch.s	*Chaetoceros simplex* Ostenf.	N	A	DIAT	17	1	0.29	1.52
Ni.sp.	*Nitzschia* spp.	M	A	DIAT	7	1	0	−0.22
Gy.s	*Gyrodinium spirale *(Bergh) Kof. & Swezy	M	M	DINO	8	1	0.38	−0.47
Da.m	*Dactyliosolen mediterraneus* (Perag.) H. Perag.	M	A	DIAT	10	1	0	0.53
Ba.d	*Bacteriastrum delicatulum* Cleve	M	A	DIAT	15	1	0.32	1.02
Ch.d	*Chaetoceros decipiens* Cleve	M	A	DIAT	17	1	0.29	1.52
Ch.sp.	*Chaetoceros* spp.	M	A	DIAT	13	1	0.14	1.02
Ch.t	*Chaetoceros teres* Cleve	M	A	DIAT	16	1	0.38	1.02
Na.sp.	*Navicula* spp.	M	A	DIAT	5	1	0	−0.72
Gu.s	*Guinardia striata *(Stolterf.) Hasle	M	A	DIAT	7	1	0	−0.22
Da.b	*Dactyliosolen blavyanus* (Perag.) Hasle	M	A	DIAT	12	1	0.15	0.78
Ps.sp.	*Pseudo-nitzschia* spp.	M	A	DIAT	4	1	0	−0.97
Oc.o	*Octactis octonaria* (Ehrenb.) Hovasse	M	A	DICTYO	7	1	0	−0.22
Cy.c	*Cylindrotheca closeterium* (Ehrenb.) Reimann & J.C. Lewin	M	A	DIAT	2	1	0	−1.47
Ha.w	*Haslea wawrikae* (Hust.) Simonsen	M	A	DIAT	8	1	0.22	−0.22
Ge.h	*Gephyrocapsa huxleyi* (Lohmann) P.Reinh	N	A	COCCO	7	2	0	0.07
He.n	*Heterocapsa nieii *(A.R. Loebl.) L.C. Morrill & A.R. Loebl	N	M	DINO	14	2	0	2.55
Gy.f	*Gyrodinium fusiforme* Kof. & Swezy	M	M	DINO	17	2	0.46	1.49
Th.b	*Thalassionema bacillare* (Heiden) Kolbe	M	A	DIAT	10	2	0.18	0.78
Pr.a	*Proboscia alata* (Brightw.) Sundstrom	M	A	DIAT	10	2	0.5	−0.64
Wa.sp.	*Warnowia* sp.	M	M	DINO	13	2	0.5	0.07
Gy.sp.	*Gymnodinium * sp.	M	M	DINO	7	2	0	0.07
Gy.m	*Gymnodinium marinum* Kent	N	M	DINO	9	2	0	0.78
Ox.m	*Oxytoxum minutum* Rampi	N	M	DINO	8	2	0	0.43
Sc.a	*Scrippsiella acuminata* complex	N	M	DINO	9	2	0	0.78
Pr.co	*Prorocentrum cordatum *(Ostenf.) J.D. Dodge	N	M	DINO	3	2	0	−1.35
Le.g	*Lebouridinum glaucum *(M.Lebour) F. Gomez, H. Takayama, D. Moreira & P.Lopez-Garcia	M	M	DINO	8	2	0.38	−0.28
Kr.t	*Kryptoperidinium triquetrum *(Ehrenb.) Tillmann, Gottschling, Elbrachter, Kusber & Hoppenrath	N	M	DINO	6	2	0	−0.28
Ox.s	*Oxytoxum scolopax* F. Stein	M	M	DINO	5	2	0	−0.64
Di.group	*Diplopsalis* group	M	H	DINO	4	2	0	−0.99
He.h	*Hemiaulus haucki* Grunow ex Van Heurck	M	A	DIAT	4	2	0	−0.99
Pr.c	*Prorocentrum compressum* (Bailey) T.H. Abe ex J.D. Dodge	M	M	DINO	9	2	0	0.78
Ph.r	*Phalacroma rotundatum* (Clap. & J. Lachm.) Kof. & J.R. Michener	M	M	DINO	3	2	0	−1.35
Ox.v	*Oxytoxum variabile* J. Schiller	N	M	DINO	4	2	0	−0.99
Ox.r	*Oxytoxum rampii* Sournia	M	M	DINO	6	2	0	−0.28

Participation coefficient and within-module-degree scores support the pattern. Size Class (M = micro-sized, N = nano-sized); Trophy (A = autotrophic; H = heterotrophic, M = mixotrophic); Taxonomy (DIAT = diatoms, DICTYO = dictyochophyceans, DINO = dinoflagellates, COCCO = coccolithophorids)

The first sub-community, typical of TIW, was dominated by micro-sized diatoms (e.g., *Guinardia flaccida*, *Dactyliosolen mediterraneus*, *Pseudo-nitzschia* spp.), with the exception of the nano-sized *Chaetoceros simplex* and one mixotrophic dinoflagellate (*Gyrodinium spirale*). This group reflected functional complementarity and diversity. The second sub-community, associated with s-AW and m-AW, was composed mainly of nano- and micro-sized mixotrophic dinoflagellates. These taxa exhibited feeding plasticity and were more numerous in peripheral network positions.

Network analysis on centrality metrics showed that the clustering coefficient was significantly higher across trophic groups (autotrophs than mixotrophs, *p *< 0.01), taxonomic groups (diatoms than dinoflagellates, *p* < 0.01) and in the first sub-community than the second one (*p* < 0.001). Many taxa (23 out of 38, 60% of the total) were ultraperipheral nodes in the network (participation coefficient = 0, [38]), that is they had co-occurrence relationships only within their own sub-community. They were more numerous in the second sub-community (15 out of 20) than in the first one (8 out of 18). Most of the taxa/groups were peripheral nodes and no species could be identified as non-hub connector (*p *> 0.62)*.*

### Microbial Metabolism

**Fig. 6:**
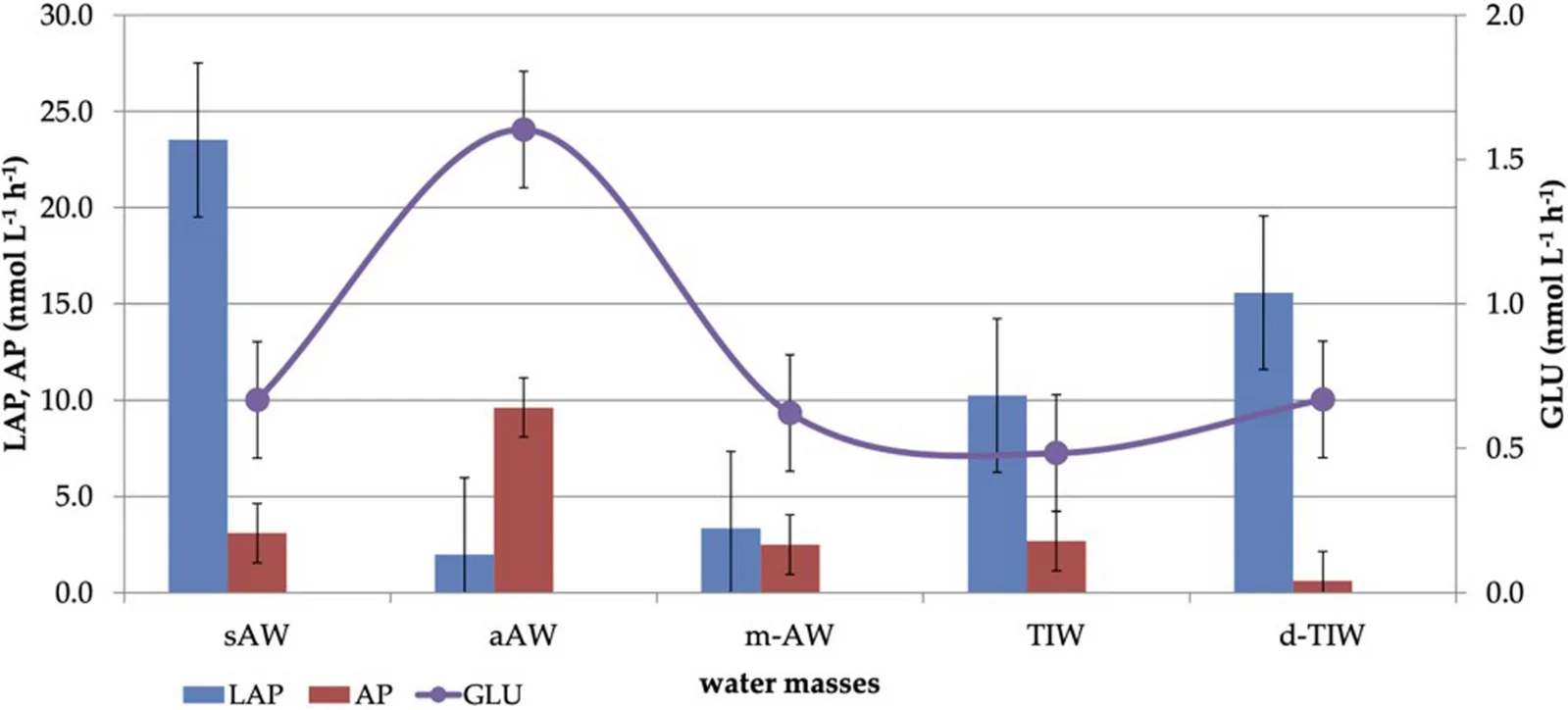
Enzyme activity rates measured across the different water masses

LAP showed the highest activity in s-AW, followed by d-TIW and TIW (Fig. 6), indicating the sinking of still-not-degraded proteins throughout the water column. In m-AW activity peaks were found at 50 m (*data not shown).* GLU activity rates reached the highest values in a-AW; lower activity rates were recorded in s-AW and m-AW (around 0.6 nmol L^−1^ h^−1^). The peak of this enzyme was recorded at 50–70 m, suggesting the presence of polysaccharides. AP distribution patterns showed that a-AW was characterized by the highest enzyme activity, followed by s-AW and lower rates in m-AW (about 1/3 of those recorded in a-AW). Within the upper DCM (u-DCM) of m-AW quick phosphorus regeneration was observed, matching with the corresponding highest nutrient consumption by phytoplankton. AP activity was at its minimum rates in d-TIW.

**Fig. 7:**
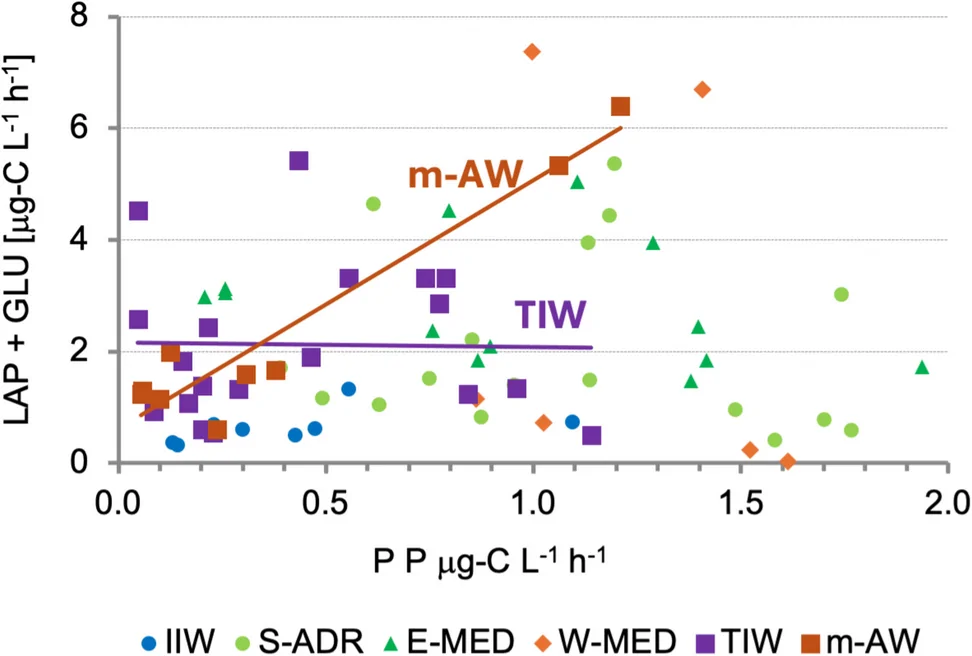
Primary production (PP) *versus* organic matter hydrolysis (LAP + GLU) in the Tyrrhenian Intermediate Waters (TIW) and mixed-Atlantic Waters (m-AW). Data from this study (TIW and m-AW) are compared with literature values from other Mediterranean regions: Ionian Intermediate Waters (IIW), Southern Adriatic Sea (S-ADR), Eastern Mediterranean Sea (E-MED), and Western Mediterranean Sea (W-MED) [12, 39]

To explore autotrophic-heterotrophic coupling, primary production was plotted against LAP + GLU hydrolysis rates at DCM (Fig. 7). In m-AW, both PP and hydrolysis increased, indicating active organic matter production and degradation, primarily of labile proteins. In contrast, in TIW, hydrolysis remained constant despite rising PP, suggesting limited microbial decomposition and potential export of unprocessed organic matter.

## Discussion

### Influence of Water Masses and Seasonality On Phytoplankton Community

Although not exhaustive, our results indicated that prevailing vertical stratification conditions influenced phytoplankton community structure and function while mesoscale dynamics affected their horizontal distribution. The mean integrated phytoplankton biomass, well below 40 mg chl-*a* m^−2^, places the Tyrrhenian Sea among the oligotrophic environments both in seasonal and spatial distribution. The size structure of the community showed the predominance of pico-phytoplankton biomass (66%), unexpectedly associated also with a high photosynthetic activity (PP = 69%). This suggests that picophytoplankton is a key component in oligotrophic environments, driving both temporal and spatial dynamics of phototrophic biomass and primary production [40] and not only a passive “unchanging background” component as previously described [41].

Several studies on Assimilation Number (AN) in oligotrophic environments have highlighted the variability of the phytoplankton functionality linked to different size fractions distribution throughout the water column, especially in relation to temperature changes [20, 42]. From our data, AN generally showed high rates due to high primary production, indicating the transfer of organic carbon towards the higher trophic levels. This condition varied according to both seasons and taxonomic composition of the phytoplankton community.

In s-AW, characterized by high summer temperatures, the highest AN of the whole region were due to biomass minima, while PP was sustained by active decomposition processes. In m-AW at u-DCM a high AN was observed in correspondence of the maximum PP, indicating an export of biomass from the system. This finding is consistent with a parallel trend shown by the autotrophic biomass and the heterotrophic decomposition and agrees with the predominant mixotrophic feeding behavior shown by dinoflagellates which dominated in these waters (i.e., *Gymnodinium* spp., *Prorocentrum* spp., *Phalacroma rotundatum*). Conversely, although poorly sampled, the Atlantic Waters entering the Tyrrhenian Sea (AW) were clearly identified by a peculiar chemical-physical signature, associated with the minimum of AN that indicates system stability, as demonstrated by a rather uniform concentration of phytoplankton biomass. The AN of TIW, where the DCM was located, was characterized by the maximum values of phytoplankton biomass (with a predominance of the micro-sized organisms, and particularly of diatoms) and suggested that the organic matter remained in the system. High LAP activity measured in TIW was consistent with the presence of a freshly produced organic matter pool [13, 24, 43]. Finally, in d-TIW below DCM the phytoplankton community showed high AN and degraded phaeopigments, both suggesting a poor adaptation of the community to the low light irradiance at this depth (around 6.0 μE m^−2^ s^−1^, Decembrini F., *personal communication*).

### Diversity of Phytoplankton Assemblages

The different hydrodynamic conditions of the Tyrrhenian Sea influenced phytoplankton in terms of abundance and biomass as well as of the assemblage composition. These communities were mainly associated with the environmental factors, and particularly to the seasonal temperature variability. A key observation was the persistent stratification of the water column, largely driven by climate-induced surface warming—a phenomenon already documented across the Mediterranean basin [44].

Microscopic analyses and chlorophyll-*a* data confirm the oligotrophic nature of the Tyrrhenian Sea, characterized by low nutrient levels and reduced phytoplankton productivity. Compared to other Mediterranean regions, both the phytoplankton abundance and carbon content in this area were significantly lower [45].

As regards the species composition, the nano-sized microalgae (often represented by undetermined flagellates), detected throughout the year, represented the characteristic feature of these oligotrophic waters, together with the pico-sized component, as the fractionated chl-*a* data demonstrated [46]. Nano-sized phytoflagellates were very common in Tyrrhenian coastal waters [47] and were represented by taxa belonging to algal classes quite phylogenetically distant, such as cryptophyte, mamiellophytes, prasinophytes, haptophytes, and pelagophytes [48].

Dinoflagellates were present with the highest number of taxa and contributed mostly to the total phytoplankton biomass in all the water masses due to their ability to move along the water column and their mixotrophic behavior [49, 50] enabling them to adapt to different light and trophic conditions.

Only approximately 20% of the identified taxa were significantly represented, forming two distinct sub-communities that primarily reflected seasonal variations.

The summer assemblage was characterized by an increase in diatoms, which are typically favored by nutrient availability and water mixing associated with the spring and autumn periods [51]. In contrast, in the Southern Tyrrhenian Sea, the summer presence of diatoms resembled coastal conditions observed in the Gulf of Naples, where recurrent blooms of small diatoms have been reported [47]. This phenomenon has been attributed to active coastal–pelagic coupling processes in the region [25]. ANOVA applied to the centrality metrics suggested that, especially in this sub-community, diatoms tend to create tight-knit associations featuring a dense network of co-occurrence links. Taxa from the genera *Chaetoceros*, *Nitzschia*, and *Pseudo-nitzschia* are characteristic of Mediterranean waters [52], and their co-occurrence has previously been observed during summer in the study area [25]. Even if in this period the number of species, abundance and biomass of diatoms increased, they were not responsible for primary production, which was supported by picophytoplankton. n-MDS analysis suggested a close association between both active phytoplankton biomass, coccolithophorids, phaeopigments, and enzymatic activities, particularly LAP; conversely, diatoms and dinoflagellates remained unclustered (*data not shown*). A similar arrangement of the phytoplankton taxa was observed in m-AW.

In the autumn period, characterized by nutrient-depleted and stratified waters associated to s-AW and m-AW, dinoflagellates contributed significantly to phytoplankton diversity with a high number of taxa, typical of a “mature” phytoplankton community [51]. In the network, many taxa co-occurred only within their own sub-community, suggesting that this latter was more “self-consistent”, more stable and consequently more mature. In warm waters, the organic matter decomposition was in an active phase supporting the phytoplankton productive activity. In m-AW, LAP was significantly related with the pico-sized chl-*a* biomass (Pearson r = 0.71, *p* < 0.05), in s-AW, GLU and AP correlated with the pico- and micro-sized fractions (r = 0.54, *p* < 0.05 respectively) suggesting the origin of these enzymes mainly produced by small-sized organisms.

### Trophic and Metabolic Role of Microbial Compartment

Microbial trophic pathways in the Central-Southern Tyrrhenian Sea reflected the system’s oligotrophic nature, as previously confirmed by other biological parameters. However, in the DCM, a shift toward a multivorous system was observed, combining microbial and nano-planktonic pathways.

**Fig. 8:**
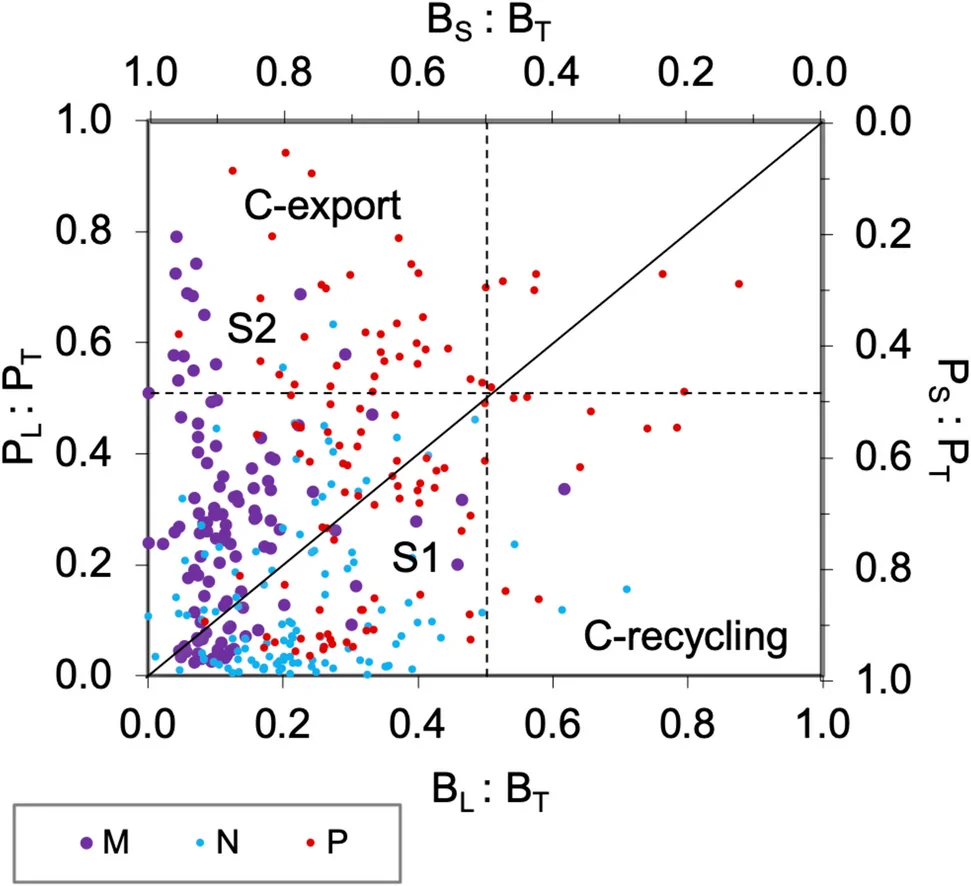
Biomass *versus* Production ratio diagram in the euphotic layer and relative importance of different size-classes (M = micro-, N = nano- and P = pico-phytoplankton) in all the samples. The regions of the plan above and below the diagonal indicate accumulation (Sector 1 below diagonal) and export processes (Sector 1 above the diagonal and Sector 2), respectively. B_S_ = Biomass of Small organisms; B_L_ = Biomass of Large organisms; B_T_ = Biomass of Total organisms; P_S_ = Production of Small organisms; P_L_ = Production of Large organisms; P_T_ = Production of Total organisms

The fate of biogenic carbon—whether recycled or exported—was assessed using production-to-biomass ratios across size fractions (Fig. 8). Most data points fell within Sector 1 (B_L_:B_T_ and P_L_:P_T_ < 0.5), indicating oligotrophy. Sector 2 (B_L_:B_T_ < 0.5 and P_L_:P_T_ > 0.5) suggested biomass export to higher trophic levels as already reported [6, 53–55]. Nano-phytoplankton predominantly occupied Sector 1, with low P_L_:P_T_ ratios (< 0.1), indicating biomass accumulation. Micro-phytoplankton showed higher P_L_:P_T_ ratios (up to 0.8), suggesting active carbon export. Pico-phytoplankton were distributed across both sectors, with notable activity in TIW and DCM, supporting their role in carbon transfer to higher trophic levels.

Enzymatic activities, particularly LAP and AP, peaked in surface and intermediate layers (s-AW and a-AW), indicating rapid organic matter turnover and efficient nutrient recycling. These activity levels were consistent with other Mediterranean regions [3, 15, 24, 56]. At greater depths, reduced enzymatic activity limited nutrient mobilization [24]. Seasonal variations, especially increased LAP in summer, reflected the presence of more labile organic matter and microbial responsiveness to environmental changes [12, 13, 24, 43, 57].

## Conclusions

In the Central-Southern Tyrrhenian Sea, water column stratification primarily shaped phytoplankton structure, function and diversity. In this oligotrophic area a relevant contribution of pico-phytoplankton to total autotrophic biomass suggested a more active role in primary production than previously assumed.

High taxonomic diversity was observed, including diatoms, dinoflagellates, coccolithophorids, and phytoflagellates. Two seasonal assemblages were identified: a summer diatom-rich community linked to active coastal–pelagic nutrient exchanges, and an autumn dinoflagellate-dominated community adapted to stratified and nutrient-poor conditions.

Enzymatic activities were associated with phytoplankton assemblages and peaked in surface waters during summer, indicating active organic matter turnover. Size-fractionated biomass and primary production revealed that pico- and nano-phytoplankton mainly contributed to within-system carbon recycling, while micro-phytoplankton drove carbon export. The relationship between production and degradation processes varied across the different water masses. These dynamics, tightly coupled with hydrography and temperature, may be amplified under future environmental conditions.

Rising sea surface temperatures and persistent thermoclines are expected to decrease nutrient availability, favoring microbial recycling rather than new production. This shift could enhance microbial food webs and reduce the prevalence of larger phytoplankton, potentially leading to a decline in high-value fish resources and an overall impoverishment of the Tyrrhenian ecosystem. Our early 2000 s data offer a comprehensive overview of phytoplankton diversity and trophic dynamics in the understudied Central-Southern Tyrrhenian Sea, serving as a potential baseline for future research**.**

## Supplementary Information

Below is the link to the electronic supplementary material.

Supplementary file1 (DOCX 201 KB)

Supplementary file2 (DOCX 36 KB)

Supplementary file3 (JPG 358 KB)

Supplementary file4 (XLSX 18 KB)

## Declarations

### Data Availability

No datasets were generated or analysed during the current study.
