# Supplementary material for: Phytoplankton diversity and size structure in the Central-Southern Tyrrhenian Sea: implications for microbial functioning
Source: Microb Ecol. 2025 Nov 20;88:141. doi: 10.1007/s00248-025-02650-w (PMC12705777; doi:10.1007/s00248-025-02650-w)
Supplement: Supplementary file 2 [file 248_2025_2650_MOESM2_ESM.docx]

Abundace (cells x 103 L-1)

|  | Total |
| --- | --- |
| Winter | 20.5918 |
| Spring | 26.3444 |
| Summer | 36.3043 |
| Autumn | 20.9662 |

Abundance percentages

|  | diatoms | dinoflagellates | coccolithophorids | others |
| --- | --- | --- | --- | --- |
| Winter | 1.4774 | 2.67655 | 1.0172 | 15.4207 |
| Spring | 0.455493 | 5.24077 | 3.64649 | 17.0016 |
| Summer | 8.37754 | 7.06382 | 2.42974 | 18.4332 |
| Autumn | 1.81797 | 3.24873 | 1.06694 | 14.8325 |

Biomass (mg C L-1)

|  | Total |
| --- | --- |
| Winter | 5.40848 |
| Spring | 3.86654 |
| Summer | 8.8652 |
| Autumn | 2.50732 |

Biomass percentages

|  | diatoms | dinoflagellates | coccolithophorids | others |
| --- | --- | --- | --- | --- |
| Winter | 0.51002 | 4.68313 | 0.0187959 | 0.19653 |
| Spring | 0.0629028 | 3.48871 | 0.0667308 | 0.248197 |
| Summer | 2.38743 | 6.21289 | 0.0484078 | 0.216474 |
| Autumn | 1.03011 | 1.27379 | 0.0431246 | 0.160292 |

Fig. S2. Phytoplankton averaged distribution in the different seasons. Total abundances (cells x 10^3^ L^-1^) (a) and biomass (μg C L^-1^) (b); percentages of the main taxonomic groups (diatoms, dinoflagellates, coccolithophorids and other phytoflagellates) in terms of abundances (c) and biomass (d). Bars = + standard deviations.
