# Supplementary material for: Phytoplankton diversity and size structure in the Central-Southern Tyrrhenian Sea: implications for microbial functioning
Source: Microb Ecol. 2025 Nov 20;88(1):141. doi: 10.1007/s00248-025-02650-w (PMC12705777; doi:10.1007/s00248-025-02650-w)
Supplement: Supplementary file 2 — Supplementary file2 (DOCX 36 KB) [file 248_2025_2650_MOESM2_ESM.docx]

Fig. S2. Phytoplankton averaged distribution in the different seasons. Total abundances (cells x 10^3^ L^-1^) (a) and biomass (μg C L^-1^) (b); percentages of the main taxonomic groups (diatoms, dinoflagellates, coccolithophorids and other phytoflagellates) in terms of abundances (c) and biomass (d). Bars = + standard deviations.
