# Supplementary figures and images for: Phytoplankton diversity and size structure in the Central-Southern Tyrrhenian Sea: implications for microbial functioning
Source: Microb Ecol. 2025 Nov 20;88(1):141. doi: 10.1007/s00248-025-02650-w (PMC12705777; doi:10.1007/s00248-025-02650-w)

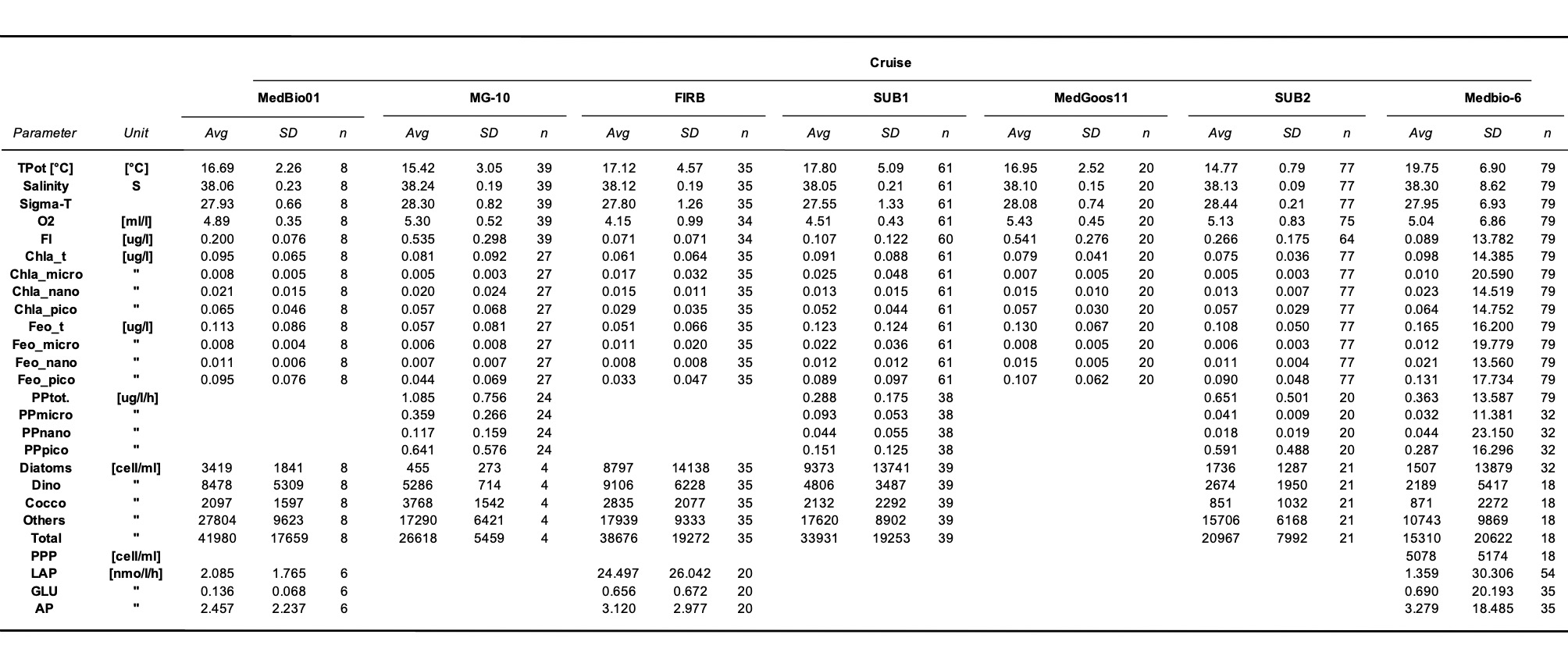

Supplement: Supplementary file 3 — Supplementary file3 (JPG 358 KB) [file 248_2025_2650_MOESM3_ESM.jpg]
